# *Caenorhabditis elegans* defective-pharynx and constipated mutants are resistant to Orsay virus infection

**DOI:** 10.17912/micropub.biology.001166

**Published:** 2024-03-24

**Authors:** Rubén González, Marie-Anne Félix

**Affiliations:** 1 Institut de Biologie de l’École Normale Supérieure-CNRS-INSERM, 75005 Paris, France.

## Abstract

*C.* *elegans* animals with a compromised pharynx accumulate bacteria in their intestinal lumen and activate a transcriptional response that includes anti-bacterial response genes. In this study, we demonstrate that animals with defective pharynxes are resistant to Orsay virus (OrV) infection. This resistance is observed for animals grown on *Escherichia coli* OP50 and on *Comamonas* BIGb0172, a bacterium naturally associated with *C. elegans* . The viral resistance observed in defective-pharynx mutants does not seem to result from constitutive transcriptional immune responses against viruses. OrV resistance is also observed in mutants with defective defecation, which share with the pharynx-defective perturbations in the regulation of their intestinal contents and altered lipid metabolism. The underlying mechanisms of viral resistance in pharynx- and defecation-defective mutants remain elusive.

**Figure 1. Defective-pharynx and constipated mutants are resistant to virus infection:**
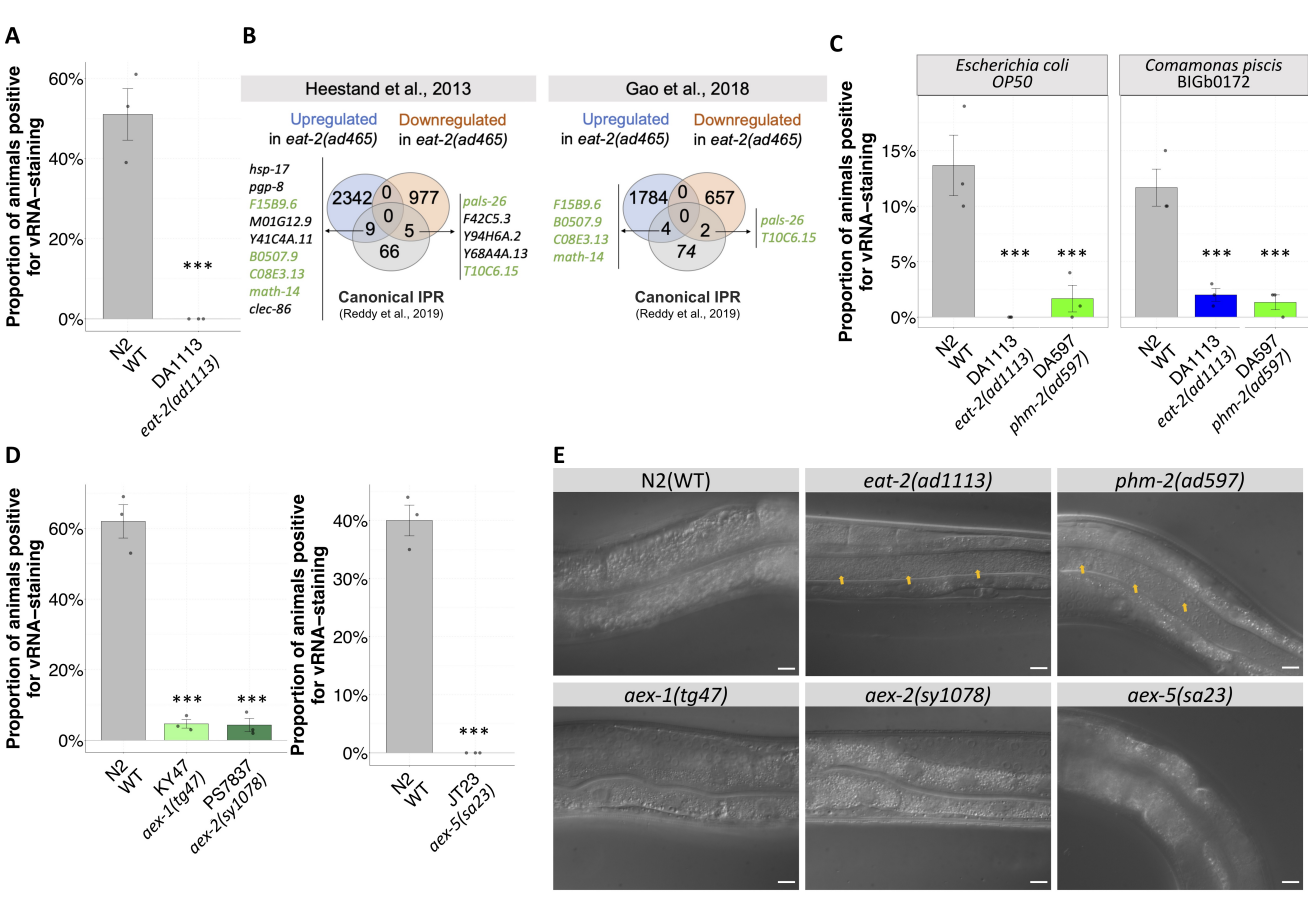
**(A)** Proportion of animals showing infected intestinal cells (stained by FISH against Orsay virus RNA2) 72 hours post-inoculation (hpi) with the virus. One experiment was performed with three biological replicates per condition. For each biological replicate, 100 animals were scored. Data are presented as mean ± standard error. Significance was calculated using a general linear model where the genotype was the fixed factor. Stars indicate the significance of the difference between the mutants and the N2 nematode strain reference (*** P < 0.001). **(B)** Venn diagram representing the overlap of differentially-expressed genes in *eat-2(ad465)* mutants and the most expressed genes of the *C. elegans* intracellular pathogen response (IPR), as defined by Reddy and colleagues (2019). The left panel uses *eat-2(ad465)* transcriptional data from Heestand and colleagues (2013) and the right one *eat-2(ad465)* data from Gao and colleagues (2018). Genes that are differentially expressed in both studies are highlighted in green text **(C)** Proportion of animals showing infected intestinal cells 72 hpi. One experiment was performed with three biological replicates per condition. For each biological replicate, 100 animals were scored. Data are presented as mean ± standard error. Significance was calculated using a general linear model where the genotype and the bacteria were the fixed factors. Tukey contrasts were used for post hoc analyses. Stars indicate the significance of the difference, in each bacterium, between the mutants and the N2 nematode strain reference (*** P < 0.001). **(D)** Proportion of animals showing infected intestinal cells 72 hpi. Panel in the left and panel in the right represent independent experiments. Each experiment was performed with three biological replicates per condition. For each biological replicate, 100 animals were scored. Data are presented as mean ± standard error. Significance was calculated using a general linear model where the genotype was the fixed factor. Tukey contrasts were used for post hoc analyses. Stars indicate the significance of the difference, in each bacterium, between the mutants and the N2 nematode strain reference (*** P < 0.001). **(E)** Nomarski micrographs of the intestine of young adults (3 days after embryo) of different genotypes cultured in *E. coli* OP50. Yellow arrows point to bacteria present in the gut. Scale bar represents 10 microns.

## Description

*Caenorhabditis elegans* is found naturally in rotting fruit and decomposing vegetal matter, environments rich in bacteria, which comprise its primary diet (Félix and Duveau, 2012; Frézal and Félix, 2015). *C. elegans* feeding is primarily governed by its pharynx, a neuromuscular pump that joins the mouth to the intestine (Avery and You, 2012) . *C. elegans* mutants with a compromised pharynx show an accumulation of live bacteria in the intestine. When exposed to *Escherichia coli* OP50 , these mutants exhibit heightened innate immunity responses and bacterial avoidance behavior (Kumar et al., 2019) .

The Orsay Virus (OrV) is a virus naturally occurring in *C. elegans* (Félix et al., 2011). This virus is a single-stranded RNA virus with a bipartite genome. OrV virions enter *C. elegans* intestinal lumen while the animal feeds and, once inside, may infect the intestinal cells (Félix and Wang, 2019). The infection reduces *C. elegans'* fitness, by reducing and delaying reproduction (Ashe et al., 2013) .

**Pharynx mutant *eat-2 ( ad1113 )* is resistant to viral infection.**

To investigate whether pharynx mutants show resistance to virus infection, we first tested *eat-2 ( ad1113 )* , a mutant with a defective pharynx. We placed axenic embryos of *eat-2 ( ad1113 )* or N2 animals (the genetic background of the mutant) in Orsay virus-inoculated plates seeded with *Escherichia coli* OP50 . Three days later, the proportion of infected individuals was assessed by FISH staining of viral RNA in the animals. We observed that in comparation with N2 , *eat-2 ( ad1113 )* animals were resistant to OrV infection ( Figure 1A ). As the presence of bacteria in the intestine can trigger immune responses, we wondered whether pharynx mutants had constitutively activated the antiviral transcriptional response. Thus, we compared the transcriptional data from *eat-2 ( ad465 )* mutants (Heestand et al., 2013; Gao et al., 2018) with the 80 canonical Intracellular Pathogen Response (IPR) genes (Reddy et al., 2019; Lažetić et al., 2022). Note that these data use animals carrying a different *eat-2* allele but also displaying a defective pharynx and intestinal colonization. In both datasets, we found little overlap with the transcriptional response against viruses ( Figure 1B ).

**Pharynx mutants are resistant to viral infection in the naturally associated bacteria *Comamonas piscis* BIGb0172.**

We wondered whether other pharynx mutants would also show resistance to viral infection. Along with *eat-2 ( ad1113 ),* we tested *phm-2 ( ad597 )* . In nature it is common to observe live bacteria in or associated with wild *C. elegans* (Félix and Duveau, 2012; Dirksen et al., 2016). We wonder whether the presence of a naturally associated bacterial strain in the intestine would trigger the resistance to viral infection. We tested the susceptibility to viral infection of the aforementioned pharynx mutants in *E. coli* OP50 and *Comamonas piscis* BIGb0172, a bacterium found associated with *C. elegans* in its natural habitats (Samuel et al., 2016) . We tested infections on *C. piscis* BIGb0172 because it is a natural bacterium and animals with an N2 genetic background grown on it have a proportion of virus-infected individuals similar to animals grown on *E. coli* OP50 (González and Félix, 2024). These two pharynx mutants were significantly more resistant to infection than N2 in both *E. coli* and *C. piscis* bacterial environments (Fig 1C).

Possible hypotheses for the mechanisms underlying the viral resistance of the pharynx mutants are: (i) activation of an immune response other than the transcriptional response; (ii) metabolic changes that hinder viral infection; (iii) reduced acquisition of virions from the media, as *eat-2* mutants have an 80% food intake in comparison with wild-type animals (Gomez-Amaro et al., 2015) ; (iv) accumulation of bacteria in the lumen blocking the entrance of virions into the intestinal cells.

**Constipated animals are resistant to viral infection.**

Mutants with a defective pharynx accumulate bacteria in their intestinal lumen. We wondered whether other mutants with perturbations in the regulation of their intestinal contents would be resistant to viral infection. Hence, we tested mutants with poor defecation (Thomas, 1989) . The defecation mutants *aex-1* , *aex-2* , and *aex-5* were resistant to OrV infection ( Figure 1D ). Note that defecation mutants do not have reduced pumping rates (Sheng et al., 2015) and that we observed that young adults accumulated fewer bacteria in the intestine than the pharynx mutants ( Figure 1E ). The mechanism behind the viral resistance of defective-pharynx and constipated mutants could in principle be different. We note lipid metabolism aberrations in both pharynx and defecation mutants (Mörck and Pilon, 2006; Sheng et al., 2015), which may be relevant for Orsay virus infections (Casorla-Perez et al., 2022) . Future investigations could focus on (i) elucidating the mechanisms underlying virus resistance in the mutants and (ii) exploring the viral dynamics within the mutants to assess potential viral clearance.

## Methods

***C. elegans* strains and maintenance**

Nematodes were cultured on Nematode Growth Media (NGM) plates at 20°C, following standard procedures (Brenner, 1974; Stiernagle, 2006) . The NGM plates were seeded with 100 µL of a monobacterial culture. The nematode strains used were N2 , DA1113 [ *eat-2 ( ad1113 )* ], DA597 [ *phm-2 ( ad597 )* ], KY47 [ *aex-1 ( tg47 )* ], PS7837 [ *aex-2 ( sy1078 )* ], JT23 [ *aex-5 ( sa23 )* ].

**Bacterial maintenance**

Bacteria were cryo-preserved at -80°C in Luria broth medium (LB; 10 g/L tryptone, 5 g/L yeast extract, 5 g/L NaCl) with 25 % glycerol. For cultivation, a cryo-preserved culture was streaked on LB-agar plates and incubated at room temperature for 48 h. Colonies were then picked and inoculated into 5 mL of liquid LB. *C. piscis* was grown at 28°C and 220 rpm for 16 h, while *E. coli* was grown at 37°C.

**Orsay virus inoculum**

The Orsay virus strain JUv1580 (Félix et al., 2011) was used in all experiments. For each experiment we defrosted and used an aliquot from a stock of viral inoculum cryo-preserved at -80°C (González and Félix, 2024).

**Virus inoculation procedure**

Nematode populations were synchronized by treating young adult populations with 4 mL of a sodium hydroxide and sodium hypochlorite mixture (2 mL sodium hypochlorite 12%, 1.25 mL NaOH 10 N, 6.75 mL H _2_ O) for three minutes. Subsequently, we washed the samples four times with 15 mL of M9 buffer. This procedure resulted in axenic embryos, which were placed around the bacterial lawn previously inoculated with 50 µL of the viral inoculum. These plates were maintained at 20°C and 72 hours post-inoculation the animals were collected to evaluate infection.

**Fluorescent in situ hybridization**

Infection was evaluated by counting the proportion of infected animals in the population. The infection on animals was visualized by staining the viral RNA in nematodes intestinal cells; the OrV RNA was stained using fluorescent in situ hybridization (FISH), following a previously described protocol (Frézal et al., 2019). In brief, animals were collected and fixed in a 10% formamide solution. Fixed animals were stained targeting OrV RNA2 molecules using a single probe (5' ACC ATG CGA GCA TTC TGA ACG TCA 3') conjugated to Texas Red.

**Microscopy**

Animals stained for OrV RNA were examined using an AxioImager M1 (Zeiss) compound microscope with 10× (0.3 numerical aperture) and 40× (1.3 numerical aperture) objectives. An animal was considered infected if at least one intestinal cell displayed distinct fluorescence at higher levels than the background staining for that individual.

Bacterial colonization of the nematodes intestinal lumen was observed with Nomarski optics with a 100× objective mounted on a Zeiss AxioSKOP microscope and captured with a CoolSNAP ES (Photometrics) camera.

**Statistical analysis**

All statistical analyses were conducted using R version 3.6.1 within the Rstudio development environment version 1.3.1093. The specific statistical methods applied to each figure are detailed in the legend of the corresponding figure.
